# Explaining hidden mechanisms: a generative model for causal graphs with nonlinear latent factors

**DOI:** 10.3389/frai.2026.1800791

**Published:** 2026-05-15

**Authors:** Koji Maruhashi, Heewon Park, Rui Yamaguchi, Satoru Miyano

**Affiliations:** 1 Fujitsu Research, Kawasaki, Kanagawa, Japan; 2 M&D Data Science Center, Institute of Integrated Research, Institute of Science Tokyo, Bunkyo City, Tokyo, Japan; 3 School of Mathematics, Statistics and Data Science, Sungshin Women's University, Seoul, Republic of Korea; 4 Human Genome Center, Institute of Medical Science, University of Tokyo, Shirokanedai, Minato City, Tokyo, Japan; 5 Division of Cancer Systems Biology, Aichi Cancer Center Research Institute, Nagoya, Aichi, Japan; 6 Division of Cancer Informatics, Nagoya University Graduate School of Medicine, Nagoya, Aichi, Japan

**Keywords:** causal abstraction, causal discovery, directed acyclic graphs, EMT, gene expression data, graph generation, identifiability, structural equation models

## Abstract

Biomedical data analysis can now recover large families of condition-specific causal graphs, yet these results are often too complex for humans to interpret mechanistically. To address this challenge, we adopt a generative perspective, we address this challenge by formulating a structural-equation-based generative model in which each observed graph is generated from a small number of latent causal mechanisms under a bidirectional causal-consistency constraint linking latent and observed causal effects. Building on this formulation, we propose Truncated Reconstruction based Interpretable Prediction (TRIP), an inference method that estimates shared latent mechanisms from observed causal graphs and condition indicators in a way that preserves interpretability across abstraction levels by learning an orthonormal projection and jointly optimizing a prediction loss and a graph-reconstruction loss. TRIP can be viewed as a supervised low-dimensional projection of graph families that makes latent mechanisms directly interpretable in terms of observed causal effects, while preserving both structural and predictive information. We validate the method on synthetic benchmarks, including a spiral-in-noise stress test, and on EMT-related gene regulatory networks derived from cancer cell-line gene expression data. Across these experiments, TRIP recovers nonlinear latent mechanisms, improves predictive performance, and organizes large condition-specific networks into interpretable mechanistic axes that can be inspected, compared, and used for hypothesis generation.

## 1 Introduction

Biomedical data analysis can now recover condition-specific causal graphs, but when these graphs are large and vary nonlinearly across conditions, the resulting structures are often too complex to interpret mechanistically. In this paper, we address the following question: given a collection of condition-specific causal graphs, can a small number of latent mechanisms be extracted to explain how and why these graphs differ across conditions? This question exposes a gap between causal discovery and scientific understanding: network estimation may succeed, yet the resulting family of graphs may remain too heterogeneous and high-dimensional to reveal its underlying mechanisms in a human-readable form.

Causal discovery has advanced rapidly in machine learning, including linear and nonlinear functional-causal approaches and differentiable structure-learning methods (Shimizu et al., 2006; Zhang and Hyvrinen, 2009; Zheng et al., 2018; Niu et al., 2024; Wang et al., 2024). Recent work has also extended causal discovery to heterogeneous and nonstationary settings in which data arise from multiple condition-specific causal graphs (Huang et al., 2020; Saeed et al., 2020; Varıcı et al., 2024; Mameche et al., 2025). Even when such methods recover varying graph structures, however, the resulting graph collections are typically too complex to provide a coherent explanation of how and why mechanisms differ across conditions. What is missing is an explicit abstraction layer that converts families of large causal graphs into a small number of mechanisms that remain interpretable to researchers.

To close this gap, a structural-equation-based generative framework is introduced in which observed condition-specific causal graphs are explained by a small number of latent causal mechanisms capturing higher-level programs shared across conditions. A key technical requirement is causal consistency (Rubenstein et al., 2017), namely, a bidirectional correspondence between latent and observed causal effects: causal relations among observed variables should be explainable through latent mechanisms, and latent causal relations should be grounded in observable effects. Building on this formulation, Truncated Reconstruction based Interpretable Prediction (TRIP) is proposed as an inference method that estimates shared latent mechanisms from observed causal graphs and condition indicators by learning an orthonormal low-dimensional projection and jointly optimizing a prediction loss and a graph-reconstruction loss. In this sense, TRIP can be viewed as a supervised projection method for graph families that preserves both structural and predictive information while converting complex graph collections into readable mechanistic axes.

**Figure 1:**
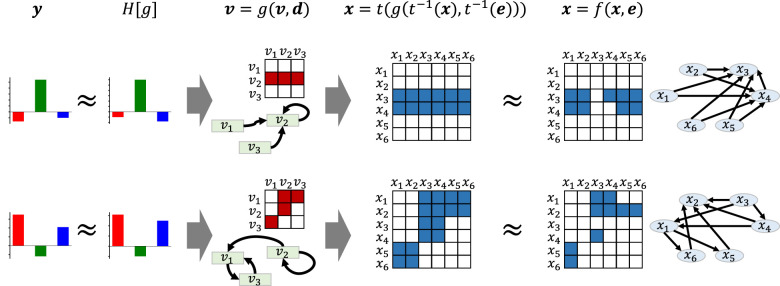
An illustration of the generative model for the causal graph. Here, *y* denotes observed indicators (labels). *H*[*g*] represents a function that maps a latent mechanism *g* to the indicator *y*. The SEM for the latent variables **v** = *g*(**v**, **d**) is generated so that the indicators **y** can be predicted as *H*[*g*] and the SEM **x** = *f*(**x**, **e**) is generated as close to **x** = *t*(*g*(*t*^−1^(**x**), *t*^−1^(**e**))) as possible. The causal graph for **x** is restricted to DAGs.

Within the framework of structural equation models (SEMs), the mapping from latent variables to observed variables is written as **x** = *t*(**v**), the latent structural mechanism as **v** = *g*(**v**, **d**), and the observed system as **x** = *f*(**x**, **e**), where **d** and **e** denote exogenous noise variables. Here, **d** and **e** denote exogenous noise variables in the SEM, capturing external influences. Causal consistency requires that the observed mechanism be approximated by **x** ≈ *t*(*g*(*t*^−1^(**x**), *t*^−1^(**e**))) so that latent mechanisms explain variation in observed graphs and remain recoverable from it. In other words, mapping observed variables into the latent space, applying the latent causal mechanism, and mapping back should reproduce the observed causal relationships, while the reverse correspondence should also hold. This conceptual relationship is illustrated in Figure 1 and motivates both the proposed generative model and the TRIP inference procedure.

The problem is studied here in the context of epithelial–mesenchymal transition (EMT), a phenotypic program closely related to cancer progression and metastasis (Haerinck et al., 2023; Zhang et al., 2025). Across EMT-related states, the same molecular interaction can exhibit different effective causal influences, producing distinct gene regulatory networks. EMT therefore provides a biologically important testbed for a broader challenge: how to interpret and compare a family of causal structures that varies with condition. More generally, however, this challenge is not specific to EMT. Whenever data analysis yields many large networks or structures, the results may remain too complex for direct interpretation even when the underlying estimation itself is technically successful.

Validation is carried out on both synthetic data and real cancer data. In synthetic experiments, TRIP is first tested for its ability to recover identifiable low-dimensional latent mechanisms from controlled graph-generation settings. A more demanding spiral-in-noise stress test is then considered, in which the essential decision boundary is a two-dimensional spiral embedded in a 100-dimensional noisy observation space, to evaluate whether a truly low-dimensional nonlinear mechanism can be recovered from high-dimensional data corrupted by nuisance variation. In the real-data analysis, gene expression profiles from 762 cancer cell lines are used, where each sample is represented by a 13,508-dimensional expression profile consisting of 13,006 mRNAs and 502 human microRNAs, together with EMT-related regulatory-network information (Shimamura et al., 2011). These experiments test whether TRIP can transform a large family of condition-specific biomedical networks into compact latent representations that are predictive, structurally grounded, and understandable to human researchers.

This study is related to causal abstraction, latent-mechanism modeling, heterogeneous causal discovery, and differentiable structure learning, but differs from these lines of research in an important respect: the goal is not only to estimate graphs, but also to recover a compact latent mechanism layer that generates, organizes, and explains graph families in an interpretable way. Causal abstraction formalizes when a higher-level model preserves the causal semantics of a lower-level system, emphasizing that interpretability requires principled correspondence across levels rather than simple compression (Rubenstein et al., 2017). In a complementary but not explicitly causal direction, latent-factor approaches such as Meta Graphical Lasso arguea that apparently complex systems may be explained by sparse dependencies among a few common latent factors across contexts (Maruhashi et al., 2024). The present work is aligned with these goals, but focuses explicitly on causal semantics: latent variables are required to explain observed graph variation through causal consistency, so that latent relations remain grounded in observable causal effects.

The framework is also complementary to recent methods for heterogeneous and nonstationary causal discovery and for scalable differentiable structure learning. Multi-environment and mixture-based approaches aim to recover component graphs, invariant relations, or edges that appear across environments (Peters et al., 2016; Huang et al., 2020; Varıcı et al., 2024; Mameche et al., 2025), while differentiable causal discovery has greatly improved the scalability and stability of large-graph estimation through continuous optimization (Zheng et al., 2018; Nazaret et al., 2024; Ma et al., 2024; Zhou et al., 2025). These advances are essential for estimating graph structure, but they do not directly solve the interpretability problem that arises once a large family of condition-specific graphs has been obtained. By contrast, the aim here is to learn a compact latent causal mechanism layer that generates, organizes, and explains such graph families, thereby enabling direct comparison across conditions in a form that remains readable to researchers.

The aim, therefore, is not simply to infer another causal graph, but to provide an abstraction layer that makes families of complex causal graphs understandable. By recovering a small number of shared latent mechanisms from observed graph collections, TRIP makes it possible to inspect, compare, and interpret condition-dependent network variation in a principled and technically explicit manner. The main contributions of this paper are threefold: (1) a generative SEM-based model that makes families of complex causal graphs interpretable through a small number of latent mechanisms; (2) an inference method, TRIP, that estimates these mechanisms from observed graphs and condition indicators through orthonormal low-dimensional projection and joint prediction–reconstruction learning; and (3) empirical validation on synthetic benchmarks and a real EMT-related cancer dataset, demonstrating recovery of nonlinear mechanisms and interpretable organization of large condition-specific networks.

## 2 Related work

### 2.1 Causal abstraction, semantic latents, and interpretable latent mechanisms

A central goal in causal modeling is to explain complex, high-dimensional systems through *higher-level* mechanisms that remain meaningful for humans. Causal abstraction formalizes when a coarse-grained model preserves causal relations of a lower-level system, emphasizing that interpretability requires principled correspondence across levels, not just compression (Rubenstein et al., 2017). This connects to causal representation learning, which seeks latent variables that correspond to stable and interpretable mechanisms. In a complementary (not explicitly causal) direction, Meta graphical lasso argues that complex systems can often be explained by sparse dependencies among a few common latent factors across contexts, enabling interpretable latent mechanism discovery (Maruhashi et al., 2024). Our work aligns with these goals but targets causal semantics: we enforce a bidirectional correspondence between latent and observed causal relations (the causal consistency ), grounding latent mechanisms in observable causal effects.

### 2.2 Heterogeneous and nonstationary mechanisms across conditions

Biomedical systems often exhibit a condition-dependent causal structure, where mechanisms vary between environments, disease subtypes, or time. Recent work addresses such heterogeneity by modeling data as mixtures of causal systems or multi-context regimes, with identifiability results and procedures for recovering edges that appear in at least one component graph or within stable contexts (Varıcı et al., 2024; Mameche et al., 2025). Related ideas in multi-environment causality leverage invariances across contexts to separate stable mechanisms from spurious associations (e.g., invariant causal prediction) (Peters et al., 2016). These approaches typically output collections of condition-specific graphs (or partially shared components); in contrast, we learn a small set of shared latent mechanisms that *generate* and organize those graphs, enabling human-readable comparison across conditions.

### 2.3 Scalable structure learning, differentiable discovery, and latent confounding

Differentiable causal discovery (DCD) enables large-scale structure estimation via continuous optimization; NOTEARS (Zheng et al., 2018) is a representative foundation. Recent variants improve stability and scalability for sparse high-dimensional graphs (Nazaret et al., 2024) and extend differentiable formulations toward constraint-based objectives via gradient-optimizable *d*-separation-style scores (Zhou et al., 2025). Robustness to latent confounding has also advanced through learning MAG; e.g., SPOT scales MAG discovery under latent confounders (Ma et al., 2024). These developments strengthen structure estimation, but they do not directly address interpretability across *graph families* of graphs; our contribution is to recover an interpretable latent mechanism layer linked to observed effects via causal consistency.

### 2.4 Positioning

Expert/LLM-guided discovery helps inject priors and refine edge-level structure over observed graphs (Ankan and Textor, 2025; Kampani et al., 2024), and benchmarks increasingly stress nonstationarity and distribution shift (Cheng et al., 2024; Ferdous et al., 2025; Stein et al., 2025). Our work aims at a complementary gap: learning a compact latent causal mechanism that (i) generates families of complex observed causal graphs and (ii) supports bidirectional human-interpretable comparison across conditions through the causal consistency.

## 3 Preliminaries

### 3.1 Notation

Scalars are denoted as lowercase letters (*a*), vectors as boldface lowercase letters (**a**), and matrices as boldface capital letters (**A**). The element *i*th of **a** is denoted as *a*_*i*_. The transposed matrix of **A** is **A**^*T*^. **I** is an identity matrix and **1**, **O** are square matrices in which all values are 1, 0. The square root of the sum of the squared values in **A** is denoted as ||**A**||_2_. The inner product is denoted as 〈·, ·〉. The multiplication and division of elements are indicated as * and ⊘, respectively. The differentials of *E* over the elements of **a** and **A** are $\frac{\partial E}{\partial \text{a}}$ and $\frac{\partial E}{\partial \text{A}}$, respectively. The covariance matrix of **a** with zero mean is *cov*(**a**) = 𝔼(**aa**^*T*^).

### 3.2 Problem definition

We focus on the linear SEMs that are commonly used in causal discovery (Shimizu et al., 2006; Zheng et al., 2018). In the linear SEMs, the DAG is represented by an *I*×*I* adjacency matrix *W* = {*w*_*ij*_} where every *w*_*ij*_ represents the connection strength from one variable *x*_*j*_ to another *x*_*i*_ in the DAG. This is described as


$$\begin{array}{l} \text{x}=\text{W}\text{x}+\text{e}, \end{array}$$ (1)

where **x** is an *I*-dimensional random vector and **e** is external influence. Without loss of generality, each variable *x*_*i*_ and *e*_*i*_ is assumed to have zero mean. *e*_*i*_ are independent of each other so that there are no latent confounding variables (Spirtes et al., 2000), where **Ω** = *cov*(**e**) is diagonal. The variances of external influence can be made 1 by transforming Equation 1 to $\Omega^{-\frac{1}{2}}\text{x}=\Omega^{-\frac{1}{2}}\text{W}\Omega^{\frac{1}{2}}\Omega^{-\frac{1}{2}}\text{x}+\Omega^{-\frac{1}{2}}\text{e}$, that is, a SEM for the variables $\Omega^{-\frac{1}{2}}\text{x}$ with adjacency matrix $\Omega^{-\frac{1}{2}}\text{W}\Omega^{\frac{1}{2}}$. We assume this transformation in the following discussion, *i.e*., *cov*(**e**) = **I**. This is introduced as a theoretical normalization to simplify the derivation. Because the transformation is given by diagonal rescaling, it preserves the support of **W**, namely the directed edge pattern, while changing only the magnitudes of coefficients. In practice, the noise covariance is generally unknown; thus, this assumption should be understood as a normalization used for theoretical convenience rather than as an operational preprocessing step applied to the supplied graph estimates.

Our problem is not inferring **W** from the observed **x** but to understand why the DAG has such a structure. We have two problems:

- **Problem 1**. Can we provide a generative model satisfying causal consistency for a causal relationship **W** corresponding to the indicators **y**?
- **Problem 2**. Given a set of causal relationships {**W**_*k*_} and corresponding indicators {**y**_*k*_}, how can we infer the parameters of the generative model?

## 4 Projecting causal structures embedded in complex data to human-interpretable dimension

### 4.1 Generative model

Our generative model is illustrated in Figure 1. Note that the mapping *t* and the functional *H* are fixed according to the domain of interest, such as cancer EMT. As our focus is the linear SEM, we provide a simple generative model where the *J*-dimensional latent variables **v** have the linear SEM **v** = **Uv**+**d** using the *J*×*J* adjacency matrix **U** and **x** is linearly related to **v** by **x** = **Cv** using the *I*×*J* matrix **C**. Note that, unlike the observed graph **W**, the latent interaction matrix **U** is not intended to represent a sparse latent DAG. Rather, **U** is a continuous-valued summary of latent mechanisms in the projected space, and it may therefore be dense. As the columns of **C** form an orthonormal basis (**C**^*T*^**C** = **I**), the latent variable **v** can be interpreted as the orthogonal projection of **x** onto the subspace spanned by **C**. We begin by considering the reconstruction problem


$$\arg\underset{\text{v}}{\min}||\text{x}-\text{C}\text{v}{||}_{2}^{2}.$$

The solution is given by the left pseudoinverse:


$$\text{v}={\left({\text{C}}^{T}\text{C}\right)}^{-1}{\text{C}}^{T}\text{x}={\text{C}}^{T}\text{x}.$$

Under the orthonormality constraint **C**^*T*^**C** = **I**, this simplifies to **v** = **C**^*T*^**x**. This relation clarifies the correspondence between the observed variable **x** and the latent variable **v** used in the following derivation.

This means **v** = **C**^*T*^**x**, and the exact transformations can be described as: **x** = **Wx**+**e** should be performed as close to **x** = **CUC**^*T*^**x**+**CC**^*T*^**e** as possible, and **v** = **Uv**+**d** should be derived by **v** = **C**^*T*^**WCv**+**d**. Both points are satisfied by **U** = **C**^*T*^**WC**, which is the optimal **U** that minimizes $||\text{W}-\text{C}\text{U}{\text{C}}^{T}{||}_{2}^{2}$.

We define the functional as *H*[**U**].

Generally speaking, there can be several mechanisms, *e.g*., mechanisms related to metabolism and immunity. Assuming there are *S*-mechanisms, **y**, **U**, **C**, and *H* are described as {**y**_*s*_}, {**U**_*s*_}, {**C**_*s*_}, and {*H*_*s*_}, respectively (*s* = 1, ⋯ , *S*). We suppose that there are no direct interactions between mechanisms, *i.e*., ${\text{C}}_{s}^{T}{\text{C}}_{s}=\text{I}$ for all *s* and ${\text{C}}_{s}^{T}{\text{C}}_{t}=\text{O}$ for all *s*≠*t*. This orthogonality assumption is introduced for interpretability, to represent latent mechanisms as separable axes. It does not imply that real biological mechanisms are independent. We propose using ${\text{U}}_{s}={\text{C}}_{s}^{T}\text{W}{\text{C}}_{s}$, the optimal **U**_*s*_ that minimizes $||\text{W}-\underset{s}{\sum }{\text{C}}_{s}{\text{U}}_{s}{\text{C}}_{s}^{T}{||}_{2}^{2}$. Moreover, **W** should be optimized so that the loss function $\underset{s}{\sum }loss\left({\text{y}}_{s},H_{s}\left[{\text{U}}_{s}\right]\right)$ is minimized. Here, we use the term “generative model” to describe a structural relationship in which observed graphs **W** are determined so as to be consistent with latent mechanisms **U** and indicators **y**. This differs from probabilistic generative models based on sampling. In summary, given the functionals *H*_*s*_ and **C**_*s*_ and the indicator **y**_*s*_, our generative model is


$$\begin{array}{l} \text{W}=\underset{\text{W}}{\text{arg min}}D\text{ subject to }G\left(\text{W}\right)\in DAGs, \end{array}$$ (2)

where


$$\begin{array}{l} D=\underset{s}{\sum }loss\left({\text{y}}_{s},H_{s}\left[{\text{U}}_{s}\right]\right)+\beta ||\text{W}-\underset{s}{\sum }{\text{C}}_{s}{\text{U}}_{s}{\text{C}}_{s}^{T}{||}_{2}^{2},\\ \;{\text{U}}_{s}={\text{C}}_{s}^{T}\text{W}{\text{C}}_{s}, \end{array}$$

Here, β is a tuning parameter that balances prediction of the indicator and reconstruction of the observed graph. It is introduced for model selection and does not correspond to a physical quantity or a “true” parameter of the data-generating process. Accordingly, our framework does not assume the existence of a true β in nature. How to choose β in a more principled manner is an important direction for future investigation. *G*(**W**) is a causal graph induced by **W**. This is an optimization problem under the DAG constraint that we can solve by leveraging the NOTEARS approach (Zheng et al., 2018), as details are shown in the Supplementary Material. NOTEARS is known to perform well under assumptions such as linear SEM, independent noise, and sufficient sample size, but may degrade under strong multicollinearity. Note that $||\text{W}-\underset{s}{\sum }{\text{C}}_{s}{\text{U}}_{s}{\text{C}}_{s}^{T}{||}_{2}^{2}$ can be written as $||\text{W}{||}_{2}^{2}-\underset{s}{\sum }||{\text{U}}_{s}{||}_{2}^{2}$.

In a special case, it is possibly supposed that observed variables are separated into “cause variables” and “effect variables”, where cause variables are never affected by others, and effect variables never affect others. In that case, the rows and columns of **W** are related to effect variables and cause variables, and there is no need for the DAG constraint for *G*(**W**). {**C**_*s*_} should be separately written as {**C**_*s*_} for effect variables and {**D**_*s*_} for cause variables, and we can use ${\text{U}}_{s}={\text{C}}_{s}^{T}\text{W}{\text{D}}_{s}$.

### 4.2 Inference method

The next question is, given the observed set of {(**W**_*k*_, **y**_*k*_)} where **y**_*k*_ is a vector of the indicators of a mechanism, how we can infer the functional *H* and the matrix **C** corresponding to the mechanism. We propose an inference method *TRIP* that finds optimal *H* and **C** for the generative process, which is described as


$$\begin{array}{l} H,\text{C}=\underset{H,\text{C}}{\text{arg min}}E\text{ subject to }{\text{C}}^{T}\text{C}=\text{I}, \end{array}$$

where


$$\begin{array}{l} E=\frac{1}{K}\underset{k}{\sum }loss\left({\text{y}}_{k},H\left[{\text{U}}_{k}\right]\right)+\beta ||{\text{W}}_{k}-\text{C}{\text{U}}_{k}{\text{C}}^{T}{||}_{2}^{2},\\ \;{\text{U}}_{k}={\text{C}}^{T}{\text{W}}_{k}\text{C}. \end{array}$$ (3)

We provide a method using stochastic gradient descent (SGD). We can update the parameters of *H*, denoted as θ, by using


$$\begin{array}{l} \frac{\partial E}{\partial \theta }=\frac{1}{K}\underset{k}{\sum }\frac{\partial loss\left({\text{y}}_{k},H\left[{\text{U}}_{k}\right]\right)}{\partial \theta }, \end{array}$$ (4)

because **θ** affects only *H*. On the other hand, **C** affects other than *H*, with the orthonormal constraint of **C**. This is the optimization over Stiefel Manifolds (Absil et al., 2007). A method that optimizes the parameters of NNs over Stiefel Manifolds is proposed (Huang et al., 2018); however, we use a simpler formulation introduced by Maruhashi et al. (2018). They use the latent **Z** of the same size as **C** and calculate **C** as a matrix that satisfies the orthonormal conditions derived from **Z**. That is, we obtain


$$\begin{array}{l} \text{Z}=\text{P}\text{S}{\text{Q}}^{T} \end{array}$$ (5)

by singular value decomposition (SVD) and set as


$$\begin{array}{l} \text{C}=\text{P}{\text{Q}}^{T}, \end{array}$$ (6)

where **P** and **Q** have left and right singular vectors as column vectors and a diagonal matrix **S** has singular values. **C** is updated by updating **Z** on the basis of $\frac{\partial E}{\partial z_{ij}}=\langle \frac{\partial E}{\partial \text{C}},\frac{\partial \text{C}}{\partial z_{ij}}\rangle$, using a derivative of **C** by *z*_*ij*_. As a result, we can calculate


$$\begin{array}{l} \frac{\partial E}{\partial \text{Z}}=\phi \left(\frac{\partial E}{\partial \text{C}},\text{Z}\right), \end{array}$$ (7)

by using


$$\begin{array}{l} \phi \left(\text{A},\text{Z}\right)=\text{P}\left[\left({\text{P}}^{T}\text{A}\text{Q}-{\text{Q}}^{T}{\text{A}}^{T}\text{P}\right)⊘\left(\text{S}1+1\text{S}\right)\right]{\text{Q}}^{T}\\ \;+\left(\text{I}-\text{P}{\text{P}}^{T}\right)\text{A}\text{Q}{\text{S}}^{-1}{\text{Q}}^{T}. \end{array}$$ (8)

Here, $\frac{\partial E}{\partial \text{C}}$ can be obtained by differentiating Equation 3, resulting in


$$\begin{array}{l} \frac{\partial E}{\partial \text{C}}=\frac{1}{K}\underset{k}{\sum }{\text{W}}_{k}\text{C}\frac{\partial E}{\partial {\text{U}}_{k}^{T}}+{\text{W}}_{k}^{T}\text{C}\frac{\partial E}{\partial {\text{U}}_{k}}, \end{array}$$ (9)

and


$$\begin{array}{l} \frac{\partial E}{\partial {\text{U}}_{k}}=\frac{\partial loss\left({\text{y}}_{k},H\left[{\text{U}}_{k}\right]\right)}{\partial {\text{U}}_{k}}-2\beta {\text{U}}_{k}. \end{array}$$ (10)

Due to space limitation, we omit how to derive Equations 7, 8 and the inferring algorithm itself.

Our inferring algorithm is shown in Algorithm 1. In Algorithm 1, the latent matrix **U**_*k*_ is handled as a continuous latent representation and is not thresholded into a sparse graph. In practical applications, the causal graphs **W**_*k*_ are often not directly observed but estimated from data using causal discovery methods. Such estimates may contain errors and instability, and this uncertainty may propagate to the inferred latent mechanisms. Therefore, the interpretability of the learned mechanisms depends on the quality of the input graphs. Addressing uncertainty in upstream graph estimation is an important direction for future work.

Algorithm 1 **TRIP.**

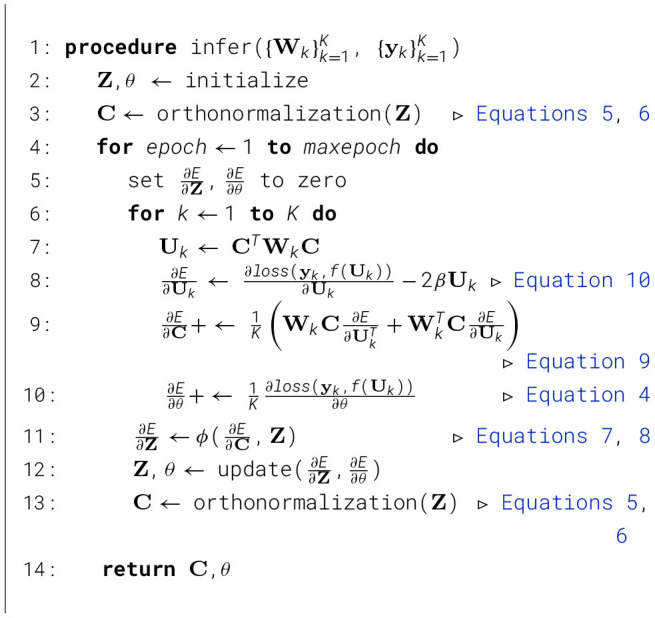

## 5 Experimental results: making nonlinear biomedical data human-interpretable

This section empirically evaluates whether TRIP can (i) recover low-dimensional latent mechanisms hidden in high-dimensional observations and (ii) present them in a form that is directly interpretable by human researchers. We report results on synthetic benchmarks—including a highly non-linear “spiral-in-noise” stress test—and on real biomedical networks derived from cancer cell-line gene expression profiles with EMT-ness scores. A key message is that rich biomedical datasets encoded biological mechanistic signals long before they were widely interpreted. TRIP recovers many EMT-related mechanisms that were only gradually uncovered in the literature over more than ten subsequent years.

### 5.1 Baselines and implementation details

Throughout this section, we use neural networks (NNs) for *H* in TRIP , using squared error loss for regression and softmax cross entropy loss for classification.

#### 5.1.1 Baselines

The most important objective of the inference method is to find the orthonormal matrix **C**. For this reason, we compare against a method similar to Tucker Decomposition (Kolda and Bader, 2009) applied to a 3rd order tensor W in which the *k*th slice along the 3rd mode is **W**_*k*_, referred to as *TuckerNN*. It finds **C** and **U**_*k*_ that minimize $\frac{1}{K}\underset{k}{\sum }||{\text{W}}_{k}-{\text{C}}^{T}{\text{U}}_{k}\text{C}{||}_{2}^{2}$ and predicts **y**_*k*_ from **U**_*k*_ using NNs. In a special case involving only one effect variable, we also compare with *PCANN* and *LDANN* , which use PCA and linear discriminant analysis (LDA) (Martínez and Kak, 2001) for converting input vectors and are combined with NNs.

#### 5.1.2 Software and hardware

All comparison methods are implemented in Python 3.7 using PyTorch 1.4^1^. All experiments were conducted on an Intel(R) Xeon(R) Gold 6130 CPU (Intel Corporation, Santa Clara, CA, United States) with 32GB of memory, running Linux.

### 5.2 Synthetic experiments

We first validate identifiability and interpretability in controlled settings. We then consider a highly non-linear scenario where the essential decision boundary is a two-dimensional spiral embedded in a 100-dimensional space with substantial noise.

### 5.3 Synthetic causal graphs

#### 5.3.1 Data generation

**Figure 2:**
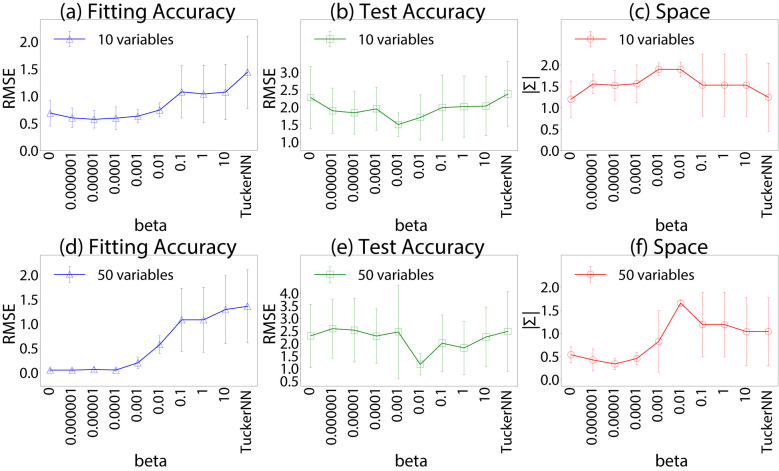
Accuracy of inference of the generative model. See Section 5 for details. **(a)** Fitting accuracy, **(b)** test accuracy, **(c)** space, **(d)** Fitting accuracy, **(e)** test accuracy, **(f)** space.

We assume two mechanisms (*S* = 2) and each mechanism involves two latent variables (*J* = 2) and three indicators (|**y**_*sk*_| = 3). The loss function is $\overset{2}{\underset{s=1}{\sum }}\overset{3}{\underset{i=1}{\sum }}||y_{sik}-H_{si}\left[{\text{U}}_{sk}\right]{||}_{2}^{2}$ where *H*_*si*_[·] is a NN with one hidden layer of 10 neurons. We conducted two experiments with different numbers of observed variables: *I* = 10 and *I* = 50. We set β = 0.001. This value was chosen to balance the two terms in the objective, whose typical scales are substantially different: the reconstruction term $||\text{W}-\text{C}\text{U}{\text{C}}^{T}{||}_{2}^{2}$ aggregates errors over all matrix entries and is typically on the order of 10^2^ to 10^3^, whereas the prediction loss for **y** is typically on the order of 10^−1^ to 10^0^. Thus, β = 0.001 gives the two terms comparable influence during optimization. This choice also yielded good predictive performance in the experiments shown in Figure 2. Each indicator in **y**_*sk*_ is sampled from a uniform distribution [−5, 5]∈ℝ. We generate **C**_*s*_ by creating matrices **Z**_*s*_ of size 10 × 2 or 50 × 2 from normal distribution N(0,1) and ${\text{C}}_{s}={\text{P}}_{s}{\text{Q}}_{s}^{T}$ obtained by SVD ${\text{Z}}_{s}={\text{P}}_{s}{\text{S}}_{s}{\text{Q}}_{s}^{T}$. All NN parameters **θ**_*s*_ are sampled from N(0,1). We leverage the NOTEARS algorithm (Zheng et al., 2018) using λ = 0.01 as the weight for the L1 constraint to obtain **W** satisfying Equation 2. We generate 100 causal graphs each for parameter inference and evaluation.

#### 5.3.2 Human-interpretable visualization of latent mechanisms

To evaluate whether the proposed generative model captures mechanisms that are strongly associated with the indicators **y**, we analyze the relationship between the latent variables **U**, the generated graphs **W**, and the indicators. A key challenge is that **U** is a matrix-valued representation, making it difficult to directly visualize multiple samples. Furthermore, the predictor *H* is nonlinear, which makes it hard to interpret how **U** relates to **y**. To address this, we approximate the nonlinear mapping *H* with a linear model for each mechanism *s* and indicator dimension *i*.

**Figure 3:**
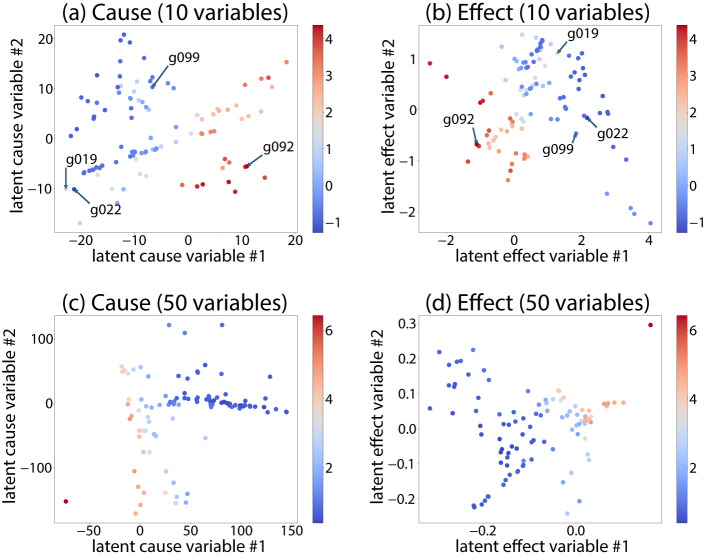
Visualization of latent mechanisms using a linear approximation of the nonlinear predictor. To interpret the relationship between the latent variables **U** and the indicator **y**, we approximate the nonlinear mapping *H* by a linear model $y_{sik}\approx {\text{g}}_{si}^{*T}{\text{U}}_{sk}{\text{h}}_{si}^{*}+b$. Each point corresponds to a graph *W*_*k*_ projected into a two-dimensional space. Figures **(a)** and **(c)** plot the first two components of ${\text{U}}_{sk}^{T}{\text{g}}_{si}^{*}$ (cause-direction aggregation), while **(b)** and **(d)** plot the first two components of ${\text{U}}_{sk}{\text{h}}_{si}^{*}$ (effect-direction aggregation). A rotation is applied so that the projected components are orthogonal. Colors indicate the indicator value *y*_*si*_. The strong alignment between the first component and *y*_*si*_ shows that the learned latent mechanisms capture variations in the indicators. The method itself is not limited to two-dimensional latent spaces; the two-dimensional plot is used only for visualization.

**Figure 4:**
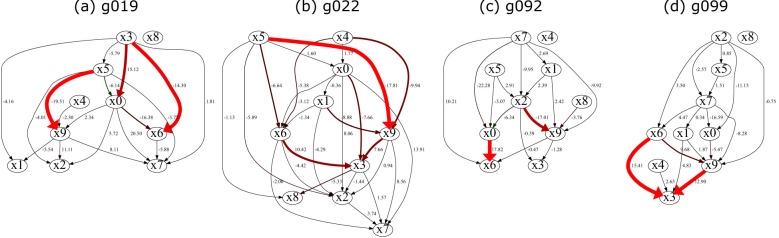
Edge-level contributions of latent mechanisms to indicator prediction. Representative graphs are selected from Figure 3a. For each graph, the contribution of each edge in **W**_*s*_ to the prediction of *y*_*si*_ is computed as ${\text{C}}_{s}{\text{g}}_{si}^{*}{\text{h}}_{si}^{*T}{\text{C}}_{s}^{T}$. Edges with positive contributions are shown in red and negative contributions in green. Graphs located close to each other in the latent space (Figure 3) exhibit similar high-contribution edges, while distant graphs do not. This demonstrates that the latent mechanisms learned by the model correspond to interpretable structural patterns in the causal graphs. **(a)** g019, **(b)** g022, **(c)** g092, **(d)** g099.

Specifically, for each sample *k*, we approximate


$$y_{sik}\approx {\text{g}}_{si}^{*T}{\text{U}}_{sk}{\text{h}}_{si}^{*}+b,$$

where ${\text{g}}_{si}^{*}$ and ${\text{h}}_{si}^{*}$ are obtained by alternating least-squares regression. This approximation allows us to interpret **U**_*sk*_ in terms of directional causal effects. In particular, ${\text{U}}_{sk}^{T}{\text{g}}_{si}^{*}$ represents the magnitude of causal effects in the “cause direction,” aggregated along the effect dimension, while ${\text{U}}_{sk}{\text{h}}_{si}^{*}$ represents the magnitude of causal effects in the “effect direction,” aggregated along the cause dimension. To improve interpretability, we apply a rotation to **U**_*sk*_ so that the components of these projected vectors become orthogonal. Importantly, such rotation does not affect the generated graph **W**, since the same rotation applied to **C**_*s*_ and **U**_*sk*_ preserves the generative model. Using this transformed representation, we visualize each graph as a point in a two-dimensional space. Figures 3a, c plot the first two components of ${\text{U}}_{sk}^{T}{\text{g}}_{si}^{*}$, while Figures 3b, d plot the first two components of ${\text{U}}_{sk}{\text{h}}_{si}^{*}$. The color of each point represents the corresponding indicator value *y*_*si*_. We observe that the first component is strongly correlated with *y*_*si*_, indicating that the latent mechanisms learned by the model capture the variation of the indicators. Although Figure 3 shows a two-dimensional visualization for interpretability, the proposed method is not restricted to two or three latent dimensions. When the latent dimension is larger, interpretability can be obtained by examining the loading structure of **C**, pairwise projections of the latent variables, and edge-level contribution patterns such as those shown in Figure 4.

To further understand how these latent mechanisms are reflected in the observed graphs, we select representative graphs from Figure 3a and analyze their edge-level contributions. Specifically, we compute the contribution of each edge in **W**_*s*_ as


$${\text{C}}_{s}{\text{g}}_{si}^{*}{\text{h}}_{si}^{*T}{\text{C}}_{s}^{T},$$

which quantifies how each edge contributes to the prediction of *y*_*si*_. Note that this contribution is invariant under the rotation applied to **C**_*s*_, as ${\text{g}}_{si}^{*}$ and ${\text{h}}_{si}^{*}$ are transformed accordingly. Figure 4 visualizes these contributions. We observe that graphs located close to each other in Figure 3a share similar high-contribution edges, while graphs located far apart do not. Positive contributions are shown in red and negative contributions in green. In this example, most large contributions are positive, and negative contributions are relatively small, resulting in few visible green edges. This demonstrates that the proposed generative model produces latent mechanisms that are both predictive of the indicators and interpretable in terms of graph structure.

#### 5.3.3 Inferring parameters

Next, we infer the parameters of the generative models by using the generated {**W**_*k*_}. Though the causal graphs are generated on the basis of 6 indicators *y*_*sik*_ of *s* = 1, 2 and *i* = 1, 2, 3, we infer parameters using only the indicator for *s* = 1, *i* = 1 with NNs with one hidden layer of 10 neurons. We conduct 10 trials with different randomly generated parameters and report the solution achieving the smallest objective value among repeated initializations within each trial.

Figures 2a, d shows that TRIP fits the indicators with lower RMSE than TuckerNN. Meanwhile, it robustly predicts the indicators of held-out evaluation data at properly chosen β (Figures 2b, e). To evaluate the similarity between the subspace spanned by the ground-truth **C**_1_ and the inferred **C**′, we compute the sum of singular values of ${\text{C}}_{1}^{T}{\text{C}}^{\prime }$ as |Σ|. Higher |Σ| implies more similar subspaces, and |Σ| = 2 indicates an identical 2-D subspace. The similarity peaks around the β values yielding the best generalization (Figures 2c, f), suggesting that TRIP recovers the intended latent mechanism under appropriate regularization.

### 5.4 Spiral-in-noise stress test: recovering a 2-D spiral hidden in 100-D

We next consider a highly non-linear situation designed to test whether TRIP can extract a truly low-dimensional non-linear mechanism from a high-dimensional observation corrupted by noise. Specifically, the essential decision boundary is distributed as a two-dimensional spiral, while the remaining dimensions contain nuisance variation (including major noise dimensions that match the spiral dimensions in variance, and many minor noise dimensions that can induce overfitting).

**Figure 5:**
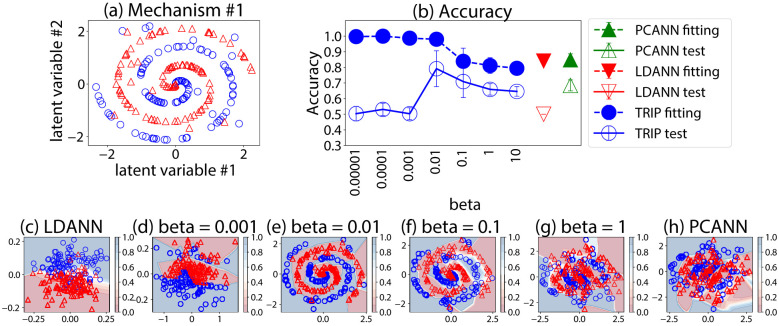
**(a)** Two-dimensional spiral distribution in the 1st mechanism. **(b)** Fitting and test accuracies. **(c–h)** Distribution of the training data projected by TRIP. Contours represent the softmax value for class 1.

We create a set of causal graphs in a special case with one effect variable and 100 cause variables, involving two mechanisms. The 1st mechanism includes two latent variables whose causal relationships with the effect variable form spiral decision boundaries (Figure 5a). The mechanism 2nd includes 98 latent variables whose relationships are randomly distributed, making the relevant signal difficult to isolate. We randomly generate **D**_1_ and **D**_2_ of size 100 × 2 and 100 × 98, respectively. Note that 1 × 1 matrices **C**_1_ and **C**_2_ are not considered. We generate 110 training samples and 110 test samples using the same procedure.

Figure 5b shows fitting and test accuracies using 2 latent variables and an NN with 3 hidden layers. The highest test accuracies are achieved at β = 0.01. Overall, smaller β causes overfitting (high fitting accuracy but low test accuracy), while overly large β leads to underfitting. The results of LDANN and PCANN resemble the low-β and high-β regimes, respectively. Figures 5c–h visualizes the mapped training samples. At appropriate β (e.g., 0.01), TRIP recovers the spiral structure in the learned 2-D representation, demonstrating that the method can make a deeply non-linear mechanism human-interpretable even when embedded in 100 dimensions.

### 5.5 Detecting and rediscovering biomedical mechanisms from EMT-ness

#### 5.5.1 Why this experiment matters

The title of this paper highlights a practical gap: biomedical datasets are often nonlinear and high-dimensional, so even when we can estimate condition-specific networks, humans struggle to extract a coherent mechanistic story. This experiment tests whether TRIP can turn a large collection of complex, cell-line–specific gene regulatory networks into a small number of *human-readable* latent mechanisms that summarize how regulation changes with a relevant cancer phenotype.

#### 5.5.2 EMT-ness as a continuous cancer phenotype score

Epithelial cells form tightly connected sheets (apical–basal polarity) that line surfaces and act as barriers for protection, absorption, and secretion, while mesenchymal cells are loosely organized, lack strong polarity, and are typically migratory, contributing to extracellular matrix production, tissue remodeling, and wound repair. The epithelial–mesenchymal transition (EMT) occurs when cancer cellular behavior changes to invasiveness and metastasis.

Rather than treating EMT as a binary label, we assigned each sample a continuous *EMT-ness score* (hereafter *EMT value*), summarizing where the sample lies along an EMT spectrum. We used the data set of the gene expression profile of 762 cancer cell lines provided by the Sanger Cell Line Project at the time of 2010 (currently, the data are expanded and included in one of the databases at the Sanger Institute). For each cancer cell line, the expression data (numerical data) of 13,006 mRNAs and 502 human microRNAs. In total, 13, 508 × 762 is the space we explore. It is so high dimensional and complex. In Shimamura et al. (2011), we defined the *EMT-ness score* as a numerical score. We first selected 50 known genes related to EMT from the literature and databases and defined the *EMT value* by combining the gene expression levels of 50 genes.

In our setting, each cancer cell line has an EMT value, and the goal is to understand how gene regulation differs across this spectrum.

#### 5.5.3 Data: a family of large networks, one per cell line

We use an EMT-status specific gene regulatory network dataset based on varying coefficients ( VC ) between 13, 508 *target genes ( TG )* (effect variables) and 1, 732 *regulator genes ( RG )* (cause variables) across 762 cell lines (Shimamura et al., 2011). Intuitively, each cell line comes with its own directed network describing how regulators (RGs) influence targets (TGs), and the strength of each relationship can vary with the EMT value. These networks are extremely large (more than 10^4^ nodes if viewed as a single unified system) and sparse; to focus on robust relationships, we set VC values with absolute magnitude less than 0.3 to zero, resulting in 2, 118 non-zero elements on average. This is precisely the kind of object that is hard for humans to interpret directly: hundreds of networks, each with thousands of nonzero edges, and a phenotype score attached to each network.

#### 5.5.4 What TRIP does (in plain terms)

Given many networks {**W**_*k*_} and their EMT values {**y**_*k*_}, TRIP learns: (i) a low-dimensional *latent mechanism space* (through **C** and ${\text{U}}_{k}={\text{C}}^{T}{\text{W}}_{k}\text{C}$) and (ii) a predictor *H* that maps each projected network to its EMT value. This has two advantages for interpretability:

- It compresses each large network into a small number of coordinates (latent variables) that can be plotted and inspected.
- It keeps the latent representation grounded in the original networks via the reconstruction term, so latent axes correspond to stable patterns in observed regulation rather than arbitrary features.

#### 5.5.5 Predictive accuracy (sanity check)

**Figure 6:**
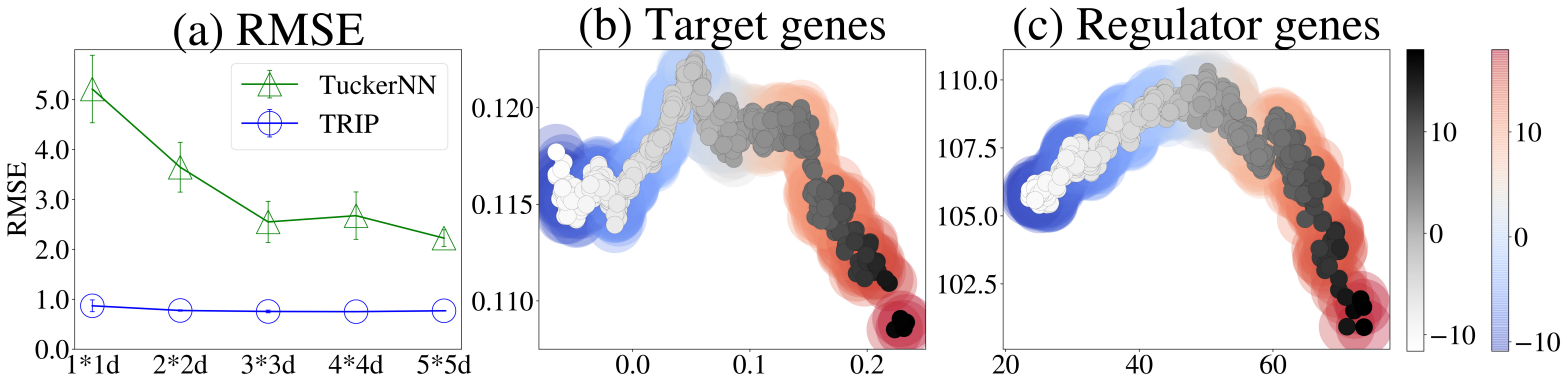
**(a)** RMSE of TuckerNN and TRIP on the gene expression data using 1 × 1- to 5 × 5 latent variables. **(b, c)** Values of data points on the 1st (horizontal) and 2nd (vertical) latent variables by TRIP. The colors indicate the EMT values, and the background colors indicate the predicted EMT values.

Figure 6a reports the RMSE of predicting EMT values. TRIP substantially outperforms TuckerNN , indicating that the learned latent mechanisms capture EMT-related variation better than purely reconstruction-driven baselines.

#### 5.5.6 Human-interpretable structure in the learned latent space

Figures 6b, c visualizes cell lines after projection onto two latent variables. Even for readers without biological background, the key point is that the projected space is *structured* and *nonlinear*: samples with similar EMT values occupy coherent regions, and the relationship between the two latent axes changes across the space. This suggests that EMT-ness is not explained by a single linear direction, but by multiple interacting patterns of regulatory change that become visible only after an appropriate mechanism-oriented projection.

#### 5.5.7 From latent axes to candidate mechanisms (which genes matter)

To translate latent axes into concrete hypotheses, we identify the most influential genes on each axis by listing TG and RG with the largest absolute loadings in the rotated **C** (Table 1). Because the sign of coefficients can be unstable under outer-product parameterizations, we focus on absolute magnitudes. We further analyze the top-50 genes using DAVID^2^ Huang et al. (2009) to obtain functional annotations that help interpret the axes. The resulting annotations differ across axes, supporting the view that the method separates distinct biological programs that contribute to EMT in different ways.

**Table 1:** Genes with high absolute scores.

Target # 1			Target # 2			Regulator # 1			Regulator # 2		
Score	Gene	t_e	Score	Gene	t_e	Score	Gene	t_e	Score	Gene	t_e
0.084	C17orf39		0.025	GTF2H3	EY	0.128	NCOR1	EP	0.097	DIP2A	UT
0.083	SLC6A8		0.025	KIAA*		0.118	IKZF1		0.035	m451	
0.072	MPP1	TM	0.024	TIGD1L		0.110	LYL1		0.034	SAP*	MS, UT
0.067	PLOD3		0.023	TCF7		0.109	DIP2A		0.032	HSF1	MS
0.062	CHKA		0.022	LOC*		0.090	AIP		0.032	NAT8	CT
0.058	SASH1	TR	0.022	PRR11	EY	0.089	SOX10		0.032	m422a*	
0.055	AFTPH		0.022	ERN2		0.088	m223		0.031	LMCD1	
0.054	GSPT1		0.022	MBTD1	EY	0.086	ELF3		0.030	TAF12	
0.053	MSX2		0.021	MTRF1L	EY	0.086	TTF1	EP	0.030	LBA1	
0.051	COASY	PL	0.021	UBQLN4	OV	0.081	CREB3		0.030	NOTCH2	

PL, platelet; TR, thyroid; TM, thymus; OV, ovary; EY, eye; EP, epithelium; MS, muscle; UT, uterus; and CT, colon tumor.

KIAA*, KIAA0894; LOC*, LOC100272216; m223, hsa-miR-223; m451, hsa-miR-451; SAP*, SAP30BP; NAT8, NAT8B; m422a*, hsa-miR-422a*.

#### 5.5.8 Rediscovery: why this supports the paper's message

After 2010, EMT biology has been actively studied, and many EMT-related regulatory mechanisms were gradually reported over the following decade. When we analyze the historical network collection through EMT-ness using TRIP , many patterns consistent with those later findings appear as salient components in the learned latent mechanisms (e.g., among top-ranked regulators/targets and axis-specific gene groups). This implies that the older, complex network data already encoded important EMT-related signals, yet those signals were not easily readable to humans directly from the raw networks. In this sense, TRIP functions as a bridge between nonlinear biomedical data and human interpretation: it converts a family of large, condition-specific networks into a small number of mechanistic axes that can be inspected, compared, and used to generate testable hypotheses.

#### 5.5.9 More on rediscovery and predictions

Further rediscoveries and predictions for EMT mechanisms by TRIP are comprehensively exhibited in Park et al. (2020) with the methodological strategy presented in this paper. This paper presents the details of mathematical methodology for the first time.

## 6 Conclusion

Nonlinear biomedical data can encode rich, condition-dependent mechanisms, yet the resulting causal structures—especially as a *family* of graphs across conditions—are often too complex for humans to interpret and compare. We argue that what is missing is an explicit *human-interpretable abstraction layer*: a small set of latent mechanisms that both organizes cross-condition variation and remains grounded in observable causal effects.

We introduced a structural-equation-based generative model that satisfies the bidirectional interpretability requirement (the causal consistency ), together with an inference method that recovers shared latent mechanisms from observed graphs and condition indicators while enforcing an orthonormal projection. Experiments show that TRIP recovers meaningful low-dimensional mechanisms even under strong nonlinearity (e.g., the spiral-in-noise stress test) and produces compact latent representations that predict EMT-ness and summarize regulatory variation in large biomedical networks.

Most importantly, our results suggest a broader implication: biomedical datasets may “know” important mechanisms long before they become human-interpretable. By converting hundreds of large condition-specific networks into a small number of readable mechanistic axes, TRIP can accelerate hypothesis generation and support condition-aware causal reasoning. Future work includes extending beyond linear SEMs and linear mappings while preserving causal consistency , modeling interactions between mechanisms, and integrating temporal/clinical contexts and intervention data to strengthen decision support.

## Data Availability

The source code for TRIP and the experiments in this study is publicly available at: https://github.com/marucozy/explaining-hidden-mechanisms. All data used in this study are either publicly available or generated as described in the manuscript.

## Footnotes

1
https://pytorch.org/

2
https://david.ncifcrf.gov/

## References

[B1] Absil P.-A. Mahony R. Sepulchre R. (2007). Optimization Algorithms on Matrix Manifolds. Princeton, NJ: Princeton University Press. doi: 10.1515/9781400830244

[B2] Ankan A. Textor J. (2025). “Expert-in-the-loop causal discovery: Iterative model refinement using expert knowledge,” in Proceedings of the forty-first conference on uncertainty in artificial intelligence, volume 286 of proceedings of machine learning research, eds. S. Chiappa, and S. Magliacane (PMLR), 172–183.

[B3] Cheng Y. Wang Z. Xiao T. Zhong Q. Suo J. He K. . (2024). “Causaltime: realistically generated time-series for benchmarking of causal discovery,” in Proceedings of the 12th International Conference on Learning Representations.

[B4] Ferdous M. H. Hossain E. Gani M. O. (2025). “Timegraph: synthetic benchmark datasets for robust time-series causal discovery,” in *Proceedings of the ACM SIGKDD Conference on Knowledge Discovery and Data Mining (KDD)*. Datasets and Benchmarks Track. arXiv [preprint]. arXiv:2506.01361.

[B5] Haerinck J. Goossens S. Berx G. (2023). The epithelial-mesenchymal plasticity landscape: principles of design and mechanisms of regulation. Nat. Rev. Genet. 24, 590–609. doi: 10.1038/s41576-023-00601-0 37169858

[B6] Huang B. Zhang K. Zhang J. Ramsey J. D. Sanchez-Romero R. Glymour C. . (2020). Causal discovery from heterogeneous/nonstationary data. J. Mach. Learn. Res. 21, 1–53.34305477

[B7] Huang D. W. Sherman B. T. Lempicki R. A. (2009). Systematic and integrative analysis of large gene lists using DAVID bioinformatics resources. Nat. Protoc. 4, 44–57. doi: 10.1038/nprot.2008.211 19131956

[B8] Huang L. Liu X. Lang B. Yu A. W. Wang Y. Li B. . (2018). “Orthogonal weight normalization: solution to optimization over multiple dependent stiefel manifolds in deep neural networks,” in Proceedings of the AAAI Conference on Artificial Intelligence (Palo Alto, CA: AAAI Press), 3271–3278. doi: 10.1609/aaai.v32i1.11768

[B9] Kampani S. Hidary D. van der Poel C. Ganahl M. Miao B. (2024). LLM-initialized differentiable causal discovery. arXiv [preprint]. arXiv:2410.21141.

[B10] Kolda T. G. Bader B. W. (2009). Tensor decompositions and applications. SIAM Rev. 51, 455–500. doi: 10.1137/07070111X

[B11] Ma P. Ding R. Fu Q. Zhang J. Wang S. Han S. . (2024). Scalable differentiable causal discovery in the presence of latent confounders with skeleton posterior (extended version). arXiv [preprint]. arXiv:2406.10537.

[B12] Mameche S. Cornanguer L. Ninad U. Vreeken J. (2025). “Spacetime: causal discovery from non-stationary time series,” in Proceedings of the AAAI Conference on Artificial Intelligence (Palo Alto, CA: AAAI Press). doi: 10.1609/aaai.v39i18.34136

[B13] Martínez A. M. Kak A. C. (2001). PCA versus LDA. IEEE Trans. Pattern Anal. Mach. Intell. 23, 228–233. doi: 10.1109/34.908974

[B14] Maruhashi K. Kashima H. Miyano S. Park H. (2024). Meta graphical lasso: uncovering hidden interactions among latent mechanisms. Sci. Rep. 14:18105. doi: 10.1038/s41598-024-68959-7 39103384 PMC11300637

[B15] Maruhashi K. Todoriki M. Ohwa T. Goto K. Hasegawa Y. Inakoshi H. . (2018). “Learning multi-way relations via tensor decomposition with neural networks,” in Proceedings of the AAAI Conference on Artificial Intelligence (Palo Alto, CA: AAAI Press), 3770–3777. doi: 10.1609/aaai.v32i1.11760

[B16] Nazaret A. Hong J. Azizi E. Blei D. (2024). “Stable differentiable causal discovery,” in Proceedings of the 41st international conference on machine learning, volume 235 of proceedings of machine learning research, eds. R. Salakhutdinov, Z. Kolter, K. Heller, A. Weller, N. Oliver, J. Scarlett (PMLR), 37413–37445.

[B17] Niu W. Gao Z. Song L. Li L. (2024). Comprehensive review and empirical evaluation of causal discovery algorithms for numerical data. arXiv [Preprint] arXiv:2407.13054. doi: 10.48550/arXiv.2407.13054

[B18] Park H. Maruhashi K. Yamaguchi Rui Imoto S. Miyano S. (2020). Global gene network exploration based on explainable artificial intelligence approach. PLoS ONE 15:e0241508. doi: 10.1371/journal.pone.0241508 33156825 PMC7647077

[B19] Peters J. Bühlmann P. Meinshausen N. (2016). Causal inference by using invariant prediction: Identification and confidence intervals. J. R. Stat. Soc. B: Stat. Methodol. 78, 947–1012. doi: 10.1111/rssb.12167

[B20] Rubenstein P. K. Weichwald S. Bongers S. Mooij J. M. Janzing D. Grosse-Wentrup M. . (2017). “Causal consistency of structural equation models,” in Proceedings of the Conference on Uncertainty in Artificial Intelligenc.

[B21] Saeed B. Panigrahi S. Uhler C. (2020). “Causal structure discovery from distributions arising from mixtures of DAGs,” in Proceedings of the 37th International Conference on Machine Learning (PMLR), 8336–8345.

[B22] Shimamura T. Imoto S. Shimada Y. Hosono Y. Niida A. Nagasaki M. . (2011). A novel network profiling analysis reveals system changes in epithelial-mesenchymal transition. PLoS ONE 6:e20804. doi: 10.1371/journal.pone.0020804 21687740 PMC3110206

[B23] Shimizu S. Hoyer P. O. Hyvärinen A. Kerminen A. J. (2006). A linear non-gaussian acyclic model for causal discovery. J. Mach. Learn. Res. 7, 2003–2030. doi: 10.5555/1248547.1248619

[B24] Spirtes P. Glymour C. Scheines R. (2000). Causation, Prediction, and Search, Second Edition. Adaptive Computation and Machine Learning. Cambridge, MA: MIT Press. doi: 10.7551/mitpress/1754.001.0001

[B25] Stein G. Shadaydeh M. Blunk J. Penzel N. Denzler J. (2025). Causalrivers-scaling up benchmarking of causal discovery for real-world time-series. arXiv [preprint]. arXiv:2503.17452.

[B26] Varıcı B. Katz D. A. Wei D. Sattigeri P. Tajer A. (2024). “Interventional causal discovery in a mixture of DAGs,” in Advances in Neural Information Processing Systems.

[B27] Wang L. Huang S. Wang S. Liao J. Li T. Liu L. (2024). A survey of causal discovery based on functional causal model. Eng. Appl. Artif. Intell. 133:108258. doi: 10.1016/j.engappai.2024.108258

[B28] Zhang C. X. Huang R. Y.-J. Sheng G. Thiery J. P. (2025). Epithelial-mesenchymal transition. Cell 188, 5436–5486. doi: 10.1016/j.cell.2025.08.033 41043405

[B29] Zhang K. Hyvärinen A. (2009). “On the identifiability of the post-nonlinear causal model,” in Proceedings of the Conference on Uncertainty in Artificial Intelligence, 647–655.

[B30] Zheng X. Aragam B. Ravikumar P. Xing E. P. (2018). “DAGs with no tears: continuous optimization for structure learning,” in Advances in Neural Information Processing Systems, 9492–9503.

[B31] Zhou J. Wang M. He A. Zhou Y. Olya H. Kocaoglu M. . (2025). “Differentiable constraint-based causal discovery,” in Advances in Neural Information Processing Systems.

